# The Influence of Serotonin on Fear Learning

**DOI:** 10.1371/journal.pone.0042397

**Published:** 2012-08-03

**Authors:** Catherine Hindi Attar, Barbara Finckh, Christian Büchel

**Affiliations:** 1 Department of Systems Neuroscience, University Medical Center Hamburg-Eppendorf, Hamburg, Germany; 2 Department of Clinical Chemistry, University Medical Center Hamburg-Eppendorf, Hamburg, Germany; University of New South Wales, Australia

## Abstract

Learning of associations between aversive stimuli and predictive cues is the basis of Pavlovian fear conditioning and is driven by a mismatch between expectation and outcome. To investigate whether serotonin modulates the formation of such aversive cue-outcome associations, we used functional magnetic resonance imaging (fMRI) and dietary tryptophan depletion to reduce brain serotonin (5-HT) levels in healthy human subjects. In a Pavlovian fear conditioning paradigm, 5-HT depleted subjects compared to a non-depleted control group exhibited attenuated autonomic responses to cues indicating the upcoming of an aversive event. These results were closely paralleled by reduced aversive learning signals in the amygdala and the orbitofrontal cortex, two prominent structures of the neural fear circuit. In agreement with current theories of serotonin as a motivational opponent system to dopamine in fear learning, our data provide first empirical evidence for a role of serotonin in representing formally derived learning signals for aversive events.

## Introduction

Learning to predict and respond to biologically relevant events is crucial to adequately adapt in a rapidly changing environment. Models of reinforcement learning (RL) are a quantitative framework for probing how the brain learns to improve predictions of future events by using a prediction error (PE) signal, representing the difference between predicted and actual outcome [1]. Previous animal recording studies have shown that midbrain dopamine neurons encode reward PEs by demonstrating increased activity at the delivery of unexpected rewards [2]. Consequently, functional MRI studies in humans that used RL algorithms highlight reward PE related activity in target regions of the mesolimbic dopamine system such as the ventral striatum (VS) and prefrontal areas including the orbitofrontal cortex (OFC) [3], [4].

Contrary to the established role of dopamine in appetitive motivation, the function of serotonin in reward and fear learning is still elusive. Besides a recent report showing a specific effect of serotonin on reward processing [5], empirical evidence has accumulated pointing towards a major role of serotonin in aversive processing [6], [7], [8], [9]. Previous studies on classical fear conditioning [10], [11] observed amygdala related signal changes decaying over the experiment, which might reflect the decrease of a prediction error signal as the association is learned. In addition, there is more recent evidence [12] suggesting a role of the amygdala in signalling aversive prediction errors associated with monetary loss.

This could be consistent with theoretical accounts [13], [14] suggesting that serotonin serves as an opponent signal to the dopaminergic reward prediction error signal by encoding a prediction error rule for aversive events. However, based on the functional diversity within neurotransmitter populations, a number of findings are difficult to reconcile with this view. For example, subpopulations of dorsal raphe (DRN) serotonin neurons were phasically excited by noxious stimuli whereas others were inhibited [15]. To further examine the role of serotonin in aversive learning we used functional magnetic resonance imaging (fMRI) and a classical aversive Pavlovian paradigm [16] together with a well-established amino-acid depletion approach [17]. Volunteers had to learn the association of visual stimuli and the occurrence of aversive thermal events. The stimulation was performed on skin pretreated with capsaicin which results in a slight irritation comparable to a sun-burn. Consequently, slight temperature increase worsens the burning sensation and is perceived as an aversive event (UCS).

By fitting a temporal difference reinforcement learning model (TDRL) to the behavioural data we observed that subjects with decreased levels of serotonin (acute tryptophan depletion [ATD], TRP-) in comparison to a non-depleted control group (TRP+) showed reduced aversive prediction error signals in the amygdala and the OFC. This was paralleled by attenuated expectancy ratings and autonomic responses to the onset of the aversive conditioned stimuli (CS+) in the 5-HT depleted group.

## Materials and Methods

### Ethics Statement

All subjects provided written informed consent prior to the study which was approved by the ethics committee of the Ärztekammer (General Medical Council) Hamburg.

### Subjects

Fifty-eight healthy, right-handed male subjects (mean age ± SD, 27.95±5.40) initially participated in this study. Two further subjects were excluded because of technical or compliance failures. From the remaining sample, 19 subjects were assigned to a third group in which explored the effect of a combined Tyrosine/Phenylalanine depletion protocol which will be reported elsewhere. Thus, the final sample included 39 subjects, with 19 subjects in the TRP+ group and 20 subjects in the TRP- group. All participants were financially compensated for participating.

### Stimuli and Pre-experimental Set-up

Stimuli consisted of phasic temperature increases (exacerbations of pain) or decreases (relief of pain, see Seymour et al., 2005). Tonic pain was induced by pretreating an area (9 cm^2^) of the skin on the left forearm with Capsaicin, the active ingredient of chilli pepper (1% Capsaicin solution: 8-Methyl-N-Vanillyl-6-Nonenamide, Sigma, diluted in 70% ethyl alcohol) leading to increased temperature sensitivity comparable to a slight sun-burn like feeling. Temperature stimuli were delivered with an overlying fMRI-compatible Peltier thermode (30×30 mm Peltier device, TSAII; Medoc) that matched the capsaicin-treated area. The day before scanning, subjective pain ratings for increases, baseline and decreases of pain were determined by using a 0–10 magnitude scale where 0 is no pain at all, and 10 is the most imaginable state of pain or relief of pain. Temperatures rated as 6/10 on the pain magnitude scale were chosen as experimental baseline temperature. For the phasic temperature increases and decreases, temperature levels of at least 8/10 indicating ‘just tolerable’ pain or a ‘fairly pleasant’ experience for the pain relief condition were chosen.

### Task

**Figure 1:**
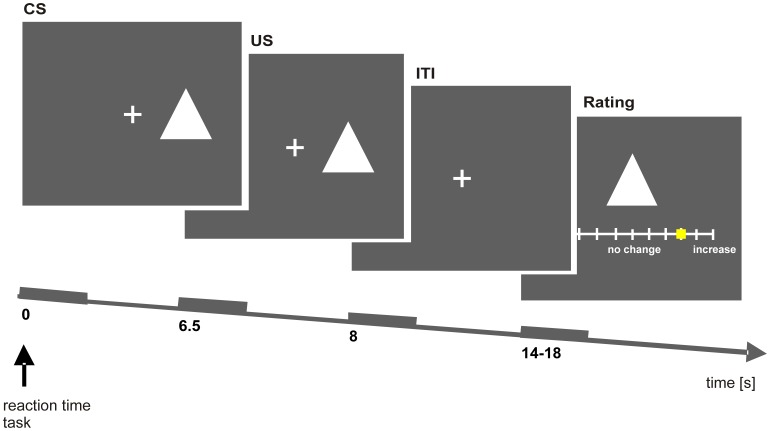
Experimental design. Each trial started with the presentation of a visual cue (simple geometric figure) as the conditioned stimulus (CS) that was presented for 8 s. 4.5 s after the onset of the cue a short phasic (1.5 s) temperature increase, decrease or no change in temperature was applied. At the beginning of each trial, subjects had to perform a reaction-time task by judging whether the visual cue appeared to the left or right of the central fixation cross. After every twelfth trial, they were prompted to rate the expected change in temperature associated with each CS on a visual analogue scale (VAS).

Based on a previously established paradigm (Seymour et al., 2005) the task was a classical Pavlovian conditioning procedure in which aversive temperature increases or appetitive temperature decreases (50% reinforcement schedule) were used as unconditioned stimuli (US). Each trial started with a visual cue as the conditioned stimulus (CS) that was presented for 8 s. 4.5 s after the onset of the cue a short phasic (1.5 s) temperature increase (aversive condition), decrease (appetitive condition) or no change in temperature (neutral condition) was applied (Figure 1). Subjects performed a reaction-time task by judging whether the visual cue appeared to the left or right of the central fixation cross. After every twelfth trial, they had to rate the expected change in temperature associated with each CS on a visual analogue scale (Figure 1). Here we only report the data for the aversive conditioning (i.e. pain exacerbation) as the appetitive part of the study (i.e. pain relief) was introduced to investigate the effect of a combined Tyrosine/Phenylalanine (i.e. Dopamine precursors) depletion in an additional group of 19 volunteers.

### Computational Model

We used a temporal difference reinforcement learning model (TDRL) [1] to estimate PE responses separately for the CS and US trial components. The two learning signals were then applied as parametric regressors to the imaging data (“model based fMRI”, see below), as previously described [4], [16]. In this TDRL model, a prediction error *δ_t_*  =  *r_t_* + *γ v(s)_t_*_+1_ − *v(s)_t_* is computed at two consecutive time steps (*t*_CS_ and *t*_outcome_  =  *t*_CS_ +1), where *v* is the predicted value of the particular cue of one condition (referred to as state *s*) at time point t, and r_t_ is the outcome at time t (i.e. pain or relief) and *γ* the discount factor for future outcomes). In the present study, we set *γ  = *1. The outcome was set to +1 for pain relief (decrease of temperature), to −1 for exacerbations of pain (increase of temperature) and to 0 when no change in temperature occurred (i.e. at the time of CS onsets and at the time of expected US onsets when outcomes were omitted). The prediction error *δ* at each time point *t* is then used to update the value signal *v* of the particular cue *s*, according to *v(s)_t_*_+1_ =  *v(s)_t_* + *α δ,* with *α* being a learning rate. Assuming that the initial expectation *v* is zero we set the initial value *v(s)* to zero.

We used each subject’s expectancy rating history to estimate the learning rate which represents the only free parameter in our model. Specifically, learning rates were individually estimated for each subject within the range 0≤ *α* ≤1 by using a standard optimization procedure that minimizes the difference between the subject’s expectancy rating for the particular CS and the model predicted value signal. In contrast to Seymour et al. (2005), we estimated separate learning rates for aversive and appetitive learning.

### Amino Acid Depletion

Subjects were randomly assigned in a double-blind manner to the TRP+ (n = 19) and TRP- (n = 20) group. All subjects received a low-protein diet (12g protein) at the day before testing and then fasted overnight. In the morning of the test day a baseline sample of blood (15 ml) was obtained before the amino acid drink was given. The quantities of amino-acids in each drink were identical to those used by Cools et al. (2008). The 5-HT- group received the same amino acids as the control group except L-tryptophan. The amino acids were dissolved in 350 ml of water and flavored with cherry syrup to make the drink more palatable. L-methionine and L-cystine were given in capsules due to their unpleasant taste. Subjects reported no side effects apart from transient nausea following ingestion of the drink.

### Autonomic Measures

Skin conductance responses (SCRs) were acquired using Ag/AgCl electrodes (Red Dot monitoring electrode; 3M Health Care) placed on the thenar and hypothenar eminence of subjects’ left hands. We used a CED 2502 to amplify the skin conductance signal, a CED micro1401 mkII to digitize the signal at 100 Hz, and Spike2 software to record and store the data (all equipment by Cambridge Electronic Design). SCR data was resampled to 10 Hz, smoothed using a 1 s full-width at half-maximum Gaussian kernel and z-transformed. We analyzed second interval responses, i.e., US anticipatory responses which are generally considered to reflect learning of the contingency [18]. To exclude confounding US influences, a time window of 2–6 s after CS onset was chosen. Amplitudes were determined as the maximum in relation to a preceding minimum in the analysis interval. SCRs to the aversive cues were then calculated by subtracting the aversive amplitudes from the amplitudes of the neutral condition.

### Imaging

MRI data was acquired on a 3T scanner (Siemens TIM-TRIO) with a 32-channel head coil. A total of 850 volumes were collected during a single session and the first 4 volumes were discarded to allow for T_1_ equilibration effects. Each volume comprised 38 contiguous descending transversal slices with a voxel size of 2×2×3 mm (TR  = 2.26 s; TE  = 25 ms; no gap). An additional MPRAGE structural image was acquired for anatomical overlay (voxel size 1×1×1 mm, 240 slices). Subjects viewed the screen via a mirror placed above the top of the head coil. The imaging data was preprocessed using SPM8 (Welcome Department of Imaging Neuroscience, London). Functional images were adjusted for slice-timing, realigned to the first volume, spatially normalized to a standard EPI template (SPM8) using third-degree B-spline interpolation, and spatially smoothed with a Gaussian kernel of 8 mm full-width at half-maximum. Data analysis was performed using the general linear model (GLM). Subject-specific cue- and outcome related PEs generated by the TDRL model were used to parametrically modulate the onset regressors corresponding to the relevant time points (*t*_1_: cue time, *t*_2_: outcome time). Depending on the reinforcement schedule, the outcome related PE signals can be either positive or negative. Specifically, unexpected relief of pain and omission of exacerbation of pain both represent positive PE signals (i.e. better than expected) while unexpected exacerbation of pain and omitted pain relief are negative PEs (i.e. worse than expected). Therefore, our parametric design included six PE regressors in total, three for each condition, one cue related PE and two outcome related PEs (i.e. positive and negative), respectively. Effects of no interest included the onsets of visual cues and the onset of pain or pain relief itself. Regressors were then convolved with the canonical hemodynamic response function. For group random effects analyses, the results from each subject were taken to the second level by including the beta images for the cue- and outcome related PE regressors from each single subject in a flexible factorial design as implemented in SPM8. Nonsphericity correction was applied to correct for a possible violation of the independence assumption of the parametric regressors. For activation in areas for which we had prior hypotheses (i.e. striatum [±18 8 0], amygdala [±20 2 −26], midbrain, near the substantia nigra [±18 −12 −8], insula [±30 22 6] and OFC [±21 47 −8]), we used small volume corrections (SVCs) to correct for multiple comparisons with 10 mm spheres for cortical and 8 mm for subcortical regions centered at activation peaks based on previous studies [4], [16]. Peaks with p<0.05 family-wise error corrected (FWE) within the small volume were considered significant. For possible brain activations outside our areas of interest, the threshold was set to p<0.05 corrected for multiple comparisons (whole brain) using FWE.

### Amino Acid Measurement

EDTA-blood was collected (10 ml) before ingestion of the amino-acid drink and again 5.5 h later to determine the levels of tryptophan (TRP) and the ratio of TRP to five further large neutral amino acids (TRP/∑LNAA) which included tyrosine, phenylalanine, isoleucine, leucine and valine. Blood samples were centrifuged (10 min; 3000 g), frozen in liquid nitrogen and stored at −80°C. Each plasma sample (100 µl) was deproteinized with 5-sulphosalicylic acid (10%, w/v) and centrifuged (3000g, 10 min) after neutralization and adding of the internal standard norleucine. The amino acids in the resulting supernatant were determined using an amino acid analyser according to standard procedures (Biochrom 30, Laborservice Onken, Gründau, Germany). A cation exchange chromatography system is coupled with a detection system using post column derivatization with ophthaldialdehyde and fluorescence detection (ex: 340 nm; em: 450 nm).

## Results

### Amino Acid Manipulation

**Figure 2:**
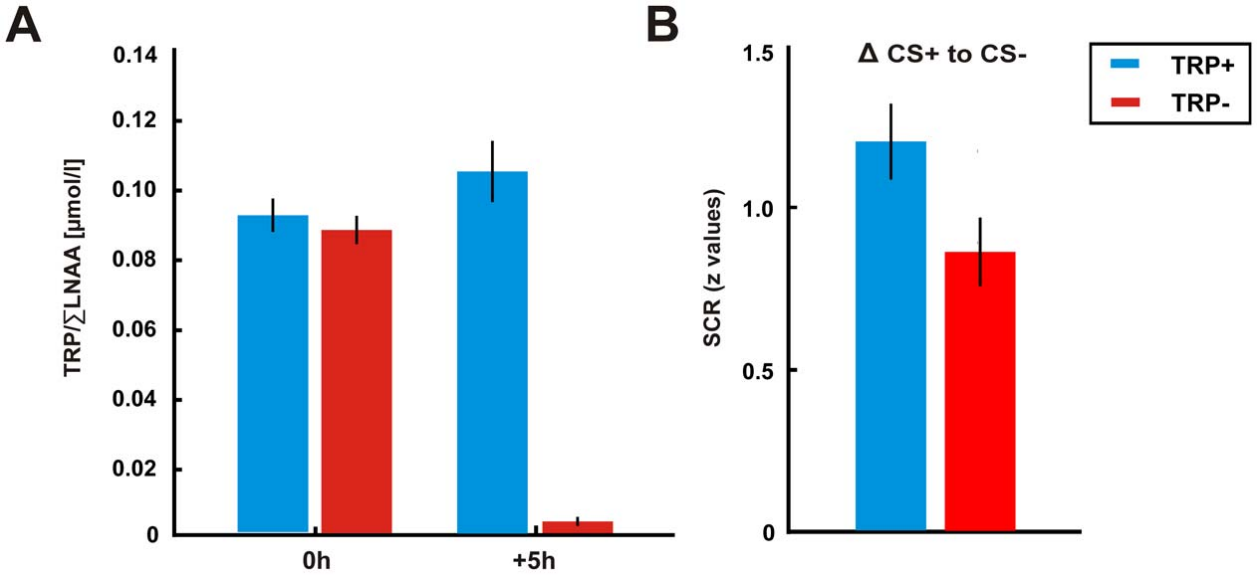
(A) Biochemical measures of tryptophan as a function of time of test and drink. The critical ratio TRP/∑LNAA measure was significantly reduced in the 5-HT depleted group (TRP-) compared to the control group (TRP+) five hours after drink ingestion. (B) Skin conductance responses (SCRs) evoked by the CS predicting an aversive outcome. SCRs of the 5-HT depleted group (TRP-) to the aversive CS+ were significantly reduced compared to the control group (TRP+). Bars represent the z-transformed SCR for each subject separately for each experimental group. Error bars represent the s.e.m.

Repeated-measures ANOVA revealed significant two-way interactions of drink by time of blood test, due to significant reductions in total tryptophan (TRP) levels (F_(1,38)_  = 161.53, p<0.0001) and in the critical ratio of TRP/∑LNAA measure (TRP/∑LNAA: F_(1,38)_  = 78.67, p<0.0001) for the 5-HT depleted group compared to the control group 5.5 h after the ingestion of amino acid mixtures (Figure 2A). Quantifying the amount of change in percent, this corresponds to a reduction of the TRP ratio of approximately 95% in the TRP- group and to a 23% enhancement in the TRP+ group, respectively.

### Behavioral and Autonomic Results

For the RT task, both groups did not differ in response latencies to the onset of the aversive CS+ as revealed by a non-significant group by condition interaction (F_1,38_ = 0.40, p>0.1). In contrast, skin conductance responses (SCRs) showed reliable CS discrimination. Specifically, a repeated-measures ANOVA comprising the factors ‘group’ and ‘condition’ yielded a significant group by condition interaction (F_1,38_ = 4.63, p<0.05). 5-HT depleted subjects compared to the control group showed a significant reduction in the SCRs to the aversive CS+ (t_38_ = 2.16, p<0.05, one-tailed; Figure 2B). An additional test of SCRs to the US revealed comparable autonomic responses across groups (group by condition: F_1,38_ = 1.27, p>0.1) thus indicating that experimental groups perceived a similar amount of pain in response to the delivery of the US.

**Figure 3:**
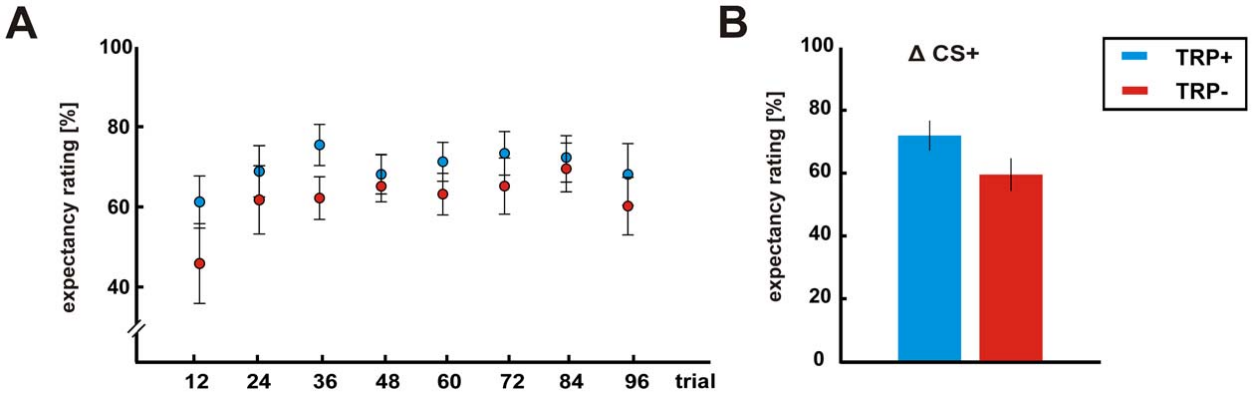
Expectancy ratings. (A) At each time point (every 12^th^ trial) subjects had to rate the expected change in temperature associated with each cue. Ratings for the aversive CS+ ranged from the least (0%) to the most certain expected (100%) pain exacerbation. (B) The right panel shows the averaged expectancy ratings for the initial learning process (i.e. first three ratings), defined as the period in which the difference between successive rating scores (averaged across treatment groups) was greater than zero (i.e. showing an increase). Averaged expectancy ratings were significantly higher for the TRP+ compared to the TRP- group. Error bars represent the s.e.m.

For the expectancy ratings (i.e. the outcome that is expected to follow each CS), both groups showed an increase in expectancy ratings for the aversive CS+. Over all time bins at which ratings were collected, TRP+ subjects yielded mean expectancy ratings of 69.61 (4.76) and TRP- subjects of 63.19 (4.58). It can be seen from Figure 3A that TRP+ compared to TRP- subjects showed an initially steeper increase. To formally capture this effect, we performed an analysis of the expectancy ratings limited to the first part reflecting the initial learning process. To define this period, we calculated the difference between rating scores from each time point (averaged across treatment groups) to the next until the difference did no longer increase (i.e. fourth rating; see Figure 3A). The average expectancy rating in the TRP+ group over the first three ratings was significantly higher than in the TRP- group (t_38_ = 1.8, p  = 0.04, one-tailed; Figure 3B).

Individual learning rates for each CS+ did not differ significantly between groups and conditions (group by condition: F_1,38_ = 0.43, p>0.1). Moreover, the average learning rate of 0.3 across groups and conditions was identical with the learning rate used previously in a similar paradigm [16].

### fMRI Results

We used a TDRL model and individual learning rates to estimate full temporal difference prediction error signals for the aversive condition at the time points of cue presentation and outcome delivery.

**Figure 4:**
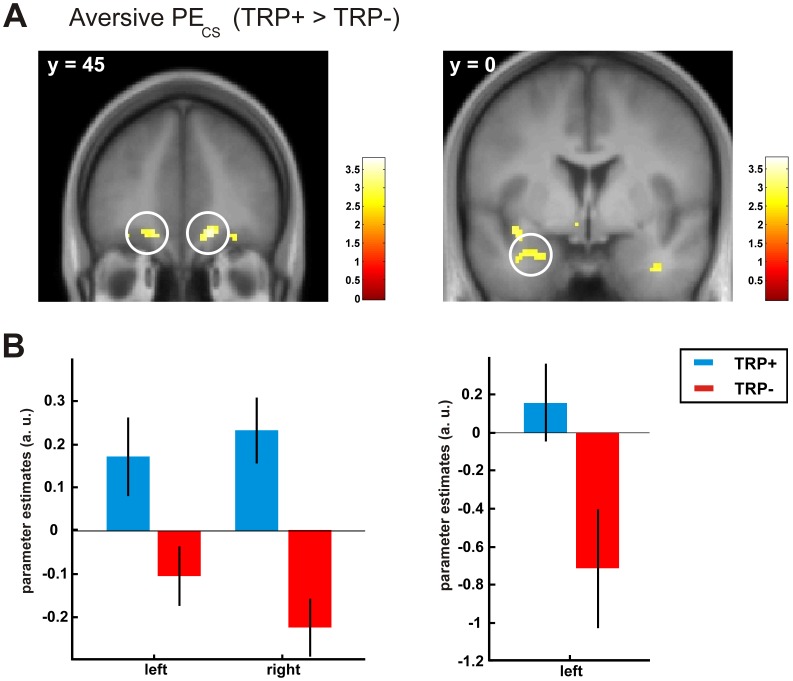
(A) Activation maps contrasting the control group with the 5-HT- group showing a significant effect for aversive prediction error signals (PE) in the orbitofrontal cortex (left) and in the amygdala (right). Activation maps are thresholded at p<0.005 (uncorrected) for visualization and are overlaid on a normalized anatomical image averaged across subjects. (B) Peak voxel parameter estimates show decreased aversive PE signals for the 5-HT depleted group (TRP-) compared to the control group (TRP+). Error bars represent the s.e.m.

At the time of cue presentation, the control group exhibited a significant main effect of the aversive PE in the OFC (14, 46, −14, Z  = 3.14, p<0.05, SVC) and in the bilateral ventral striatum (right: 14, 14, −10, Z  = 3.64, p<0.01, SVC; left: −10, 8, −8, Z  = 3.46, p<0.05, SVC). Comparisons between the experimental groups revealed that the 5-HT depleted group showed significantly decreased reductions of the aversive PE signal in the OFC (right: 14, 46, −12, Z  = 3.58, p<0.05 SVC; left: OFC (−16, 42, −14, Z  = 2.94, p  = 0.06, SVC) (Figure 4, left panel). As shown in the right panel of Figure 4, the 5-HT- group further exhibited reduced aversive PE representations in the left amygdala (−26, 2, −24, Z  = 3.15, p<0.05, SVC).

At the outcome level, we observed for the control group a significant aversive PE signals reflecting unexpected omissions of pain that correlated with the BOLD signal in midbrain regions, specifically in the midbrain, near the substantia nigra (right: 16, −10, −10, Z  = 3.6, p<0.01, SVC; left: −8, −14, −8, Z = 4.1, p<0.01, SVC). No significant group differences were observed at the outcome level.

## Discussion

Our data show that central lowering of serotonin (5-HT depletion) through dietary restrictions of the serotonin precursor tryptophan impaired aversive Pavlovian learning. 5-HT depleted subjects showed reduced PE signals in the amygdala and OFC in response to a conditioned stimulus predicting an aversive outcome which was paralleled by a similar reduction in the autonomic responses to the aversive cue.

The functional and clinical importance of serotonin is well established [19], [20], however, a formal concept comparable to the one linking the dopamine system with appetitive prediction errors, is missing [13]. This might at least in part be related to basic features of the serotonergic system which comprise a high complexity of receptor types and mechanisms leading to opposite interactions between specific 5-HT receptors, signal modes and interneuromodulator interactions [21]. Thus, it is not surprising that recordings from serotonergic compared to dopaminergic nuclei have not yet reached a similar degree of precise targeting [13].

In the present study, plasma tryptophan within the TRP- group was significantly reduced by 90% and there is good evidence from studies using positron emission tomography (PET) that this is paralleled by a central reduction in serotonin [22]. Thus, our observed effect of ATD on the aversive PE signal is most likely caused by 5-HT mediated changes [23].

We observed the strongest effect of 5-HT depletion in the OFC. Evidence for OFC 5-HT having an important influence on affective regulation comes from animal studies of reversal learning, conditioned reinforcement and extinction learning [24], [25]. The amygdala as a core region in the neural circuit of Pavlovian fear conditioning [10], [11], [26] also showed reduced aversive learning signals in the 5-HT depleted group which is in line with previous studies demonstrating prediction error related effects in the amygdala based on aversive stimuli [12], [27].

Physiological and anatomical evidence suggests the amygdala together with the OFC to be intimately involved in the valuation of stimuli [28], [29]. This has also led to an expanded conception of the amygdala’s role [30] by showing that single amygdala neurons respond differentially to a range of stimuli with positive or negative affective significance [29]. Serotonin is a key component of the neurochemical regulation of amygdala function and an abundant expression of 5-HT receptor subtypes has been found on amygdala neurons [31]. The amygdala receives dense innervation from the dorsal raphe nucleus (DRN) [32] and some recent recording studies reported reward-dependent modulation of DRN neuronal activity which further conflicts with an exclusive role of serotonin in aversion [33], [34], [35].

However, there is evidence for a role of serotonin in aversive processing which comes from behavioral studies using similar 5-HT depletion protocols [6], [8]. In the study of Crocket et al. (2009) for example, a novel reinforced go-nogo task that incorporated reward and punishment specifically abolished response slowing in punished conditions in 5-HT depleted subjects which was interpreted as a decline in aversive predictions. Together with other reports of serotonin in mediating subjects’ sensitivity to punishment [36], [37], this further challenges previous accounts of a general behavioral suppressive or inhibitory function of serotonin [38].

However, other evidence points in the opposite direction by showing that decreased levels of serotonin enhance the impact and anticipation of punishment [6]. The discrepancy might also be related to differences in the experimental paradigms. In the reversal-learning task of Cools et al. [6], the positive ATD effect was observed in the non-switch trials but not in the switch trials, which theoretically comprise larger prediction errors according to temporal difference learning [1]. In addition, rodent studies using the serotonin depletor *p*-Chlorophenylalanine showed increased responsiveness to shock deliveries and facilitated shock avoidance [39], [40]. Other studies in nonhuman primates showed that depletion of 5-HT by injection of 5,7-DHT increased perseveration in reversal learning [41], [42].

Our observed effect of 5-HT depletion on aversive learning was restricted to PE related activation at the time of CS presentation. In our paradigm, this PE signal is highly correlated with the value of the CS [43]. Importantly, we modeled both (i.e. appetitive, aversive) PEs as separate regressors at different time points (CS, US). This approach is different from Seymour et al. (2005) who aggregated all PEs in a single regressor including CS and US related PE responses and considered the aversive PE as the inverse of this regressor. Their very parsimonious model thus treats (i) pain expectation (CS), (ii) expected and delivered pain (US) and (iii) expected but omitted cooling (US) equally as an aversive prediction error, at the expense of not being able to account for different signal amplitudes for each of the three events. However, this might be important as there is evidence that CS and US related activations with respect to pain differ [44], [45].

A recent human tryptophan depletion study reported an effect of tryptophan depletion on reward outcome value representation but no similar effect on punishment learning [5]. This specificity seems contradictory to our data where we show a significant effect of ATD on the predictive learning of aversive events. However this discrepancy might be related to the nature of the task: While Seymour et al. (2012) used an operant paradigm with time-varying contingencies in which volunteers could learn to avoid aversive outcomes we employed a classical Pavlovian learning paradigm with a fixed reinforcement schedule of 50%.

Finally, it might be argued that our data could be related to a global ATD effect on blood flow rather than task-specific mechanisms on brain 5-HT levels [46]. However, Evers et al. [7] showed that ATD did not affect task-related BOLD responses during a visual control task suggesting that ATD does not induce vascular effects that lead to a global change of task-related activation. A general vascular effect is also unlikely to produce regionally restricted- and task-distinct changes in BOLD responses as observed in the present study. Thus, our findings converge with other findings from human and animal research showing reliable effects of ATD on emotional processing [23].

In summary, our study shows that the dietary reduction of serotonin impaired fear learning and the associated neural computation of aversive learning signals in the orbitofrontal cortex and the amygdala which was paralleled by attenuated expectancy ratings and autonomic responses to fear-relevant stimuli.
